# VAAST 2.0: Improved Variant Classification and Disease-Gene Identification Using a Conservation-Controlled Amino Acid Substitution Matrix

**DOI:** 10.1002/gepi.21743

**Published:** 2013-07-08

**Authors:** Hao Hu, Chad D Huff, Barry Moore, Steven Flygare, Martin G Reese, Mark Yandell

**Affiliations:** 1 Department of Epidemiology, The University of Texas M.D. Anderson Cancer CenterHouston, Texas; 2 Department of Human Genetics, Eccles Institute of Human Genetics, University of Utah School of MedicineSalt Lake City, Utah; 3 Omicia, IncEmeryville, California

**Keywords:** disease-gene finder, variant classifier, aggregative association test, rare Mendelian disease, complex disease

## Abstract

The need for improved algorithmic support for variant prioritization and disease-gene identification in personal genomes data is widely acknowledged. We previously presented the Variant Annotation, Analysis, and Search Tool (VAAST), which employs an aggregative variant association test that combines both amino acid substitution (AAS) and allele frequencies. Here we describe and benchmark VAAST 2.0, which uses a novel conservation-controlled AAS matrix (CASM), to incorporate information about phylogenetic conservation. We show that the CASM approach improves VAAST’s variant prioritization accuracy compared to its previous implementation, and compared to SIFT, PolyPhen-2, and MutationTaster. We also show that VAAST 2.0 outperforms KBAC, WSS, SKAT, and variable threshold (VT) using published case-control datasets for Crohn disease (*NOD2*), hypertriglyceridemia (*LPL*), and breast cancer (*CHEK2*). VAAST 2.0 also improves search accuracy on simulated datasets across a wide range of allele frequencies, population-attributable disease risks, and allelic heterogeneity, factors that compromise the accuracies of other aggregative variant association tests. We also demonstrate that, although most aggregative variant association tests are designed for common genetic diseases, these tests can be easily adopted as rare Mendelian disease-gene finders with a simple ranking-by-statistical-significance protocol, and the performance compares very favorably to state-of-art filtering approaches. The latter, despite their popularity, have suboptimal performance especially with the increasing case sample size.

## Introduction

Traditionally, genome-wide association studies (GWASs) have been used to identify disease-associated variants using sets of “tagging” single-nucleotide polymorphisms (SNPs) distributed across the genome. GWAS approaches, however, are underpowered to detect the effects of rare casual variants because they are usually in poor linkage disequilibrium with the tagging SNPs Visscher et al., 2012. New sequencing technologies have significantly reduced the price of human genome resequencing, and are identifying many novel rare variants. The classification and prioritization of these rare variants for disease-gene studies has thus become a significant problem.

To date, several variant prioritization tools have been developed to identify damaging alleles in personal genomes data. SIFT Ng and Henikoff, 2006 and AlignGV-GD Tavtigian et al., 2006, for example, use multiple alignments to assay conservation levels of novel amino acid changing variants with the underlying assumption that sequence variants, which alter highly conserved positions in protein sequences are a priori more likely to be damaging. Two more recently published algorithms, PolyPhen-2 Adzhubei et al., 2010 and MutationTaster Schwarz et al., 2010, improve upon this basic approach, integrating other information (e.g., protein structural changes) into the calculation, and thus significantly improving their variation prioritization accuracies compared to SIFT Ng and Henikoff, 2006.

A major weakness of many variant prioritization tools is that they can only prioritize variants within phylogenetically conserved coding regions and thus have poor coverage across the proteome. For example, SIFT and PolyPhen can score only 60% and 81% of the human proteome, respectively Adzhubei et al., 2010. Another weakness of these approaches is that they make no use of allele frequency information. It has long been known that minor allele frequency (MAF) is negatively correlated with purifying selection pressure Kryukov et al., 2007. Thus, publicly available human-genome databases (e.g., HapMap Thorisson et al., 2005, the 1000 Genomes Project (1KGP) Altshuler et al., 2010, and dbSNP Smigielski et al., 2000 provide valuable frequency information that can, in principle, be used for variant prioritization. VAAST Yandell et al., 2011 is a step forward in both regards in that it uses an approach to variant classification that combines both amino acid substitution (AAS) information with variant frequency information, allowing it to score all variants no matter where they lie in the genome and with greater accuracy Yandell et al., 2011.

The widened scope of the VAAST approach, however, comes at a cost: VAAST, in its original form, does not make any use of phylogenetic conservation data. In the present study, we describe an extension of the VAAST variant prioritization approach that makes use of a conservation-controlled AAS matrix (CASM) to overcome this shortcoming. The CASM approach allows VAAST to score every variant in the genome, and to employ phylogenetic conservation information at the same time. Our benchmark analyses presented here demonstrate that CASM approach results in the highest variant prioritization accuracies yet achieved.

Employing rare variants for disease-gene identification is another challenge. One approach is simply to search case genomes for regions having an increased density of rare variants. This is the approach taken by ANNOVAR Wang et al., 2010, which allows users to impose a threshold on variant frequencies as observed in dbSNP or in the 1000 Genomes Project Altshuler et al., 2010; Smigielski et al., 2000, excluding from further consideration variants with population frequencies above a user-defined threshold. A strength of the tool is that it can use third-party variant prioritization scores such as those produced by SIFT and PolyPhen to improve search accuracy; its principle weakness is that excluding variants with MAFs above a user-defined threshold renders the tool ineffective for searching datasets containing disease-causing alleles distributed across a range of population frequencies. In response, probabilistic approaches that overcome this limitation have emerged. These tests aggregate prioritization information from each variant in a gene to achieve greater statistical power, allowing them to bypass the need for large statistical corrections for multiple tests. These tools include CAST Morgenthaler and Thilly, 2007, CMC Li and Leal, 2008, WSS Madsen and Browning, 2009, KBAC Liu and Leal, 2010, VT Price et al., 2010, SKAT Wu et al., 2011, and VAAST Yandell et al., 2011. Although each algorithm approaches the problem differently, all either explicitly or implicitly use the MAF information to weight variants. In addition, VT and VAAST 2.0 can also use functional predictions from third-party variant prioritization tools such as PolyPhen and PhastCons Yang, 1995 to weight variants Price et al., 2010. We refer to these approaches collectively as *aggregative variant association tests*.

To date, aggregative variant association tests have been seen as a means to identify genes and variants associated with common diseases. However, the performance characteristics of different association tests as rare disease gene finders are still largely unknown. Also largely undetermined to date is the impact of factors such as population attributable risk (PAR) and allelic and locus heterogeneity on their ability to identify genes and alleles responsible for both rare and common disease Madsen and Browning, 2009.

Here we describe the new version of VAAST (VAAST 2.0) and the CASM approach. We employ a variety of datasets to benchmark VAAST 2.0, systematically comparing its performance to the original version of VAAST Yandell et al., 2011 and to other published association tests, including WSS Madsen and Browning, 2009, KBAC Liu and Leal, 2010, SKAT Wu et al., 2011, and VT Price et al., 2010. Our results demonstrate the improvements to VAAST made possible by the CASM approach; they also provide a general framework with which to investigate the performance of different aggregative variant association tests using published and simulated datasets. These results shed considerable light on the complexities involved in searching personal genomes data for disease-causing alleles as they reveal unexpected strengths and weaknesses of different approaches under different scenarios, providing a roadmap for future improvements to each method.

## Materials and Methods

### The CASM Approach

VAAST uses an extended composite likelihood ratio test (CLRT) to determine a severity score for genomic variants Yandell et al., 2011. The null model of the CLRT states that the frequency of a variant or variant group is the same in the control population (background genomes) and case population (target genomes), while the alternative model allows these two frequencies to differ. Under a binomial distribution, the likelihood for both models can be calculated based on observed allele frequencies in the control and case datasets. In VAAST 1.0 this likelihood ratio (LR) is further updated by the AAS severity parameter (*a_i_*/*h_i_*), where *h_i_* is the likelihood that an AAS does not contribute to the disease and *a_i_* is the likelihood that it does. We estimate *h_i_* by setting it equal to the frequency of this type of amino acid change in the background population, and *a_i_* by setting it equal to the frequency of the amino acid change among all disease-causing mutations in OMIM. VAAST 1.0 uses (*a_i_*/*h_i_*) to model the severity of each amino acid change. This approach, however, does not take into account phylogenetic conservation at that position of the protein, which can in theory be used to improve the accuracy of (*a_i_*/*h_i_*). In VAAST 2.0, we have extended this severity parameter by using an additional conservation measurement, PhastCons Yang, 1995 scores; these scores estimate the probability that the locus is under negative selection and are calculated using multiple species nucleotide alignments.

The CASM operates as follows: Consider first, a variant occurring at a position in the genome having some PhastCons score, and changing a valine (V) to an alanine (A). To calculate the severity parameter, we first calculate the relative frequencies of V to A causing variants at any conservation level within a disease and a nondamaging variant database. In practice, this approach is hindered by the fact that the number of such variants in the disease database may be limited. To overcome this problem, we start with estimating (*a_i_*/*h_i_*) for each type of amino acid with PhastCons scores of 0 and 1 (the two end points), as follows. For any given type of AAS *i* (*i* = 1, 2,…, *m*), suppose that there are *n_i_* variants in the disease database and each variant *j* (j = 1, 2,…, *n_i_*) has a PhastCons score of *P_ij_*. Because *P_ij_* can be interpreted as the probability that the variant is at a conserved locus Yang, 1995, the likelihood that a variant is disease causing can be estimated by
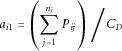
(1)for variants with a PhastCons score of 1, and
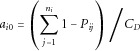
(2)for variants with a PhastCons score of 0, where *C_D_* is the total number of variants in the disease allele database used for training. Similarly using a database of nondamaging variants, the likelihood that a variant is not disease causing can be estimated by
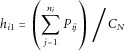
(3)for variants with a PhastCons score of 1, and
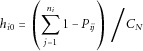
(4)for variants with a PhastCons score of 0, where *C_N_* is the total number of variants in the nondamaging allele database used for training.

Thus, the severity parameter for AAS type *i* with a PhastCons score of 0 and 1 is (*a_i_*_0_/*h_i_*_0_*)* and (*a_i_*_1_/*h_i_*_1_), respectively. For variants with other PhastCons scores (*x;* 0< *x* <1), the likelihood is estimated by a linear combination of (*a_i_*_0_/*h_i_*_0_) and (*a_i_*_1_/*h_i_*_1_), namely,
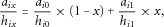
(5)where *a_ix_*/*h_ix_* are the terms in the CASM. This provides an estimate of LR of a given amino acid change being disease-causal vs. being nondamaging, controlled for the phylogenetic conservation level in the gene context. The procedures for training and testing the CASM method are detailed in the Supporting Information.

Unless otherwise noted, we calculated the severity parameter using variants from the Human Gene Mutation Database (HGMD) Cooper et al., 1998 as disease variants and using variants from the 1000 Genomes Project (Phase I data) Altshuler et al., 2010 with MAFs ^3^ 0.05 as the nondamaging variants. We first evaluated CASM on a small test dataset using PhastCons scores from three different genome alignments: UCSC vertebrate, mammal, and primate Karolchik et al., 2004. The vertebrate alignment produced the most accurate CASM scores and was used in all subsequent analyses.

### Indel Support in VAAST 2.0

VAAST 2.0 also supports small insertion and deletion (indel) mutations. The VAAST Annotation Tool, a component of VAAST package Reese et al., 2010; Yandell et al., 2011 now annotates the functional impact of indels on protein-coding genes in GVF format Reese et al., 2010. These annotations include (1) determination of whether or not the indel disrupts the reading frame of one or more protein-coding genes and if so which ones; and (2) whether the indel causes an AAS, insertion, or deletion. VAAST 2.0 then scores indels with the same CLRT as single-nucleotide variants (SNVs) that is, it calculates the LR of null model vs. alternative model for each indel variant based on its observed allele frequencies in background and target genomes, and then updates the LR with the severity parameter (*a_i_*/*h_i_*), which is estimated as follows. First, indels are classified into categories based on three properties: (1) whether it is an insertion or a deletion, (2) the affected nucleotide length, and (3) whether it disrupts the protein translation reading frame. For each category of indels, we calculate the proportion of HGMD variants falling into this category, which is our estimate of disease-causal likelihood. We also use a nondamaging variant database to determine the likelihood of being noncausal for each category. The ratio of these two likelihoods is used as (*a_i_*/*h_i_*) term to update the original LR. Note that rare indel variants are collapsed before being scored, as described in Yandell et al. 2011. This is especially important for indels, because the exact boundaries of indel variants are often called imprecisely. Collapsing variants thus allows VAAST to assess the impact of multiple overlapping indels in the cases.

## Results

### Variant Prioritization

We compared the performance of VAAST 2.0 to other variant classifiers. Whereas tools such as SIFT, PolyPhen, and Align-GD Adzhubei et al., 2010; Ng and Henikoff, 2006; Tavtigian et al., 2006 cannot score regions lacking multiple sequence alignment information, VAAST 2.0 suffers from no such limitation. In regions where no nucleotide or protein conservation data are available, VAAST 2.0 uses allele frequencies and global amino acid substation frequencies as the basis for variant prioritization; in regions where conservation information is available, VAAST 2.0 supplements this information with PhastCons scores Yang, 1995, which cover 99.9% of the human proteome. For this comparison, we limited our benchmark analysis to variants that can be scored by all four algorithms (SIFT, PolyPhen-2, MutationTaster, and VAAST 2.0). It should be kept in mind, however, that in addition to these, VAAST can also score many other variants in these datasets that the other tools cannot.

**Figure 1:**
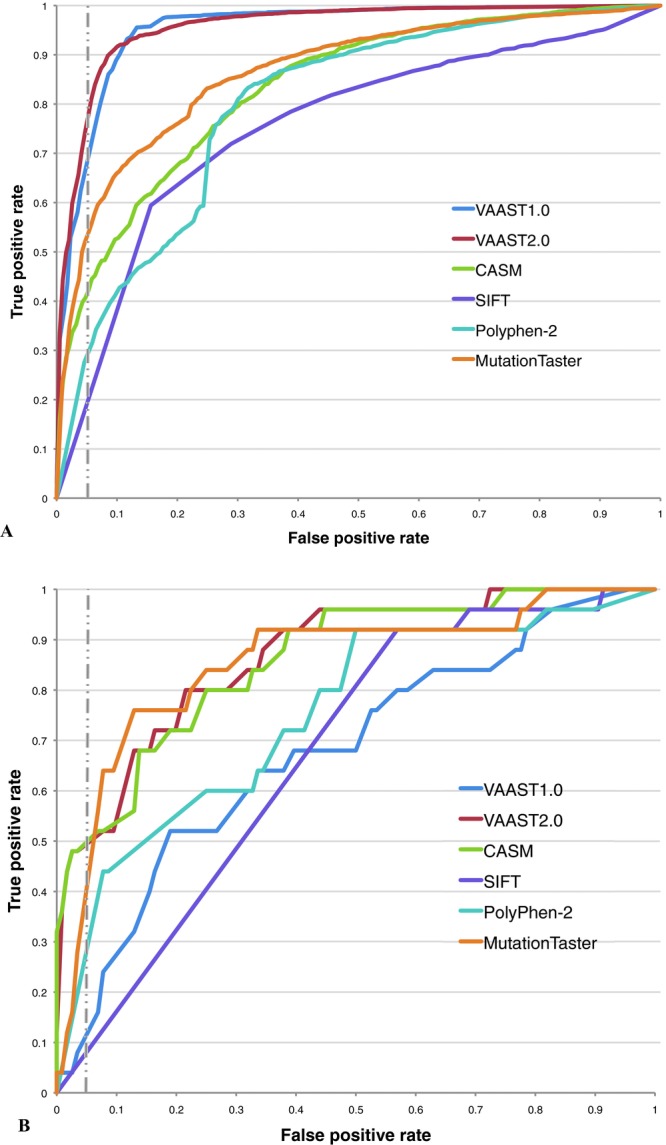
Receiver operator curves (ROC) for the variant prioritization tools. Shown are ROCs for VAAST 1.0, VAAST 2.0, CASM, SIFT, PolyPhen-2, and MutationTaster, using two benchmark datasets: (A) common and rare variants from HGMD and 1000 Genomes Project; (B) *BRCA1* and *BRCA2* rare variant set. *x*-axis: false-positive rate; *y*-axis: true positive rate. Dashed line denotes the false-positive rate of 0.05.

To evaluate the prioritization performance of each tool, we plotted the Receiver Operator Curve for each algorithm using a set of nondamaging variants (drawn randomly from the 1000 genomes project (1KGP) Pilot Phase Altshuler et al., 2010) and a set of disease-causal variants (from HGMD database) (see Supporting Information for details). Figure 1A demonstrates that the accuracy of VAAST 2.0 and 1.0 is considerably better than the other algorithms, with the true positive rate (TPR) reaching 76% for VAAST 2.0 and 68% for VAAST 1.0 when the false-positive rate (FPR) is 5%. The third best tool is MutationTaster, whose TPR is 23% lower than VAAST 2.0 at the same FPR level. VAAST 2.0 using the CASM method alone without recourse to variant frequency information (“CASM” in Fig. 1) is the fourth best performing approach, followed by PolyPhen-2 and SIFT. We also calculated the area under the curve value and the accuracy at FPR = 0.05 for each algorithm, which demonstrates the same trends (Table1).

**Table 1:** Variant prioritization performance benchmarks

	VAAST1.0	VAAST2.0	CASM	SIFT	PolyPhen-2	MutationTaster
Area under the curve (AUC)						
Dataset 1 (HGMD+1KGP)	0.95	0.96	0.83	0.76	0.8	0.87
Dataset 2 (rare *BRCA* variants)	0.68	0.87	0.86	0.73	0.76	0.85
Accuracy at FPR of 0.05						
Dataset 1 (HGMD+1KGP)	0.81	0.86	0.68	0.57	0.62	0.74
Dataset 2 (rare *BRCA* variants)	0.53	0.72	0.72	0.52	0.62	0.68

For a second variant prioritization benchmark, we compared the performance of each of these algorithms using a set of 143 rare missense variants in the *BRCA1* and *BRCA2* genes whose clinical significance was assessed by a third party Easton et al., 2007. This variant set differs from the HGMD/1KGP variants used to produce Figure 1A in that the dataset used to produce Figure 1A contains both common and rare variants for nondamaging and deleterious alleles, whereas this set Figure 1B only contains very rare variants (MAF << 1%). The results of this benchmark analysis are shown in Figure 1B and Table1. Because majority of the variants in this set are observed only once, VAAST 2.0 cannot use the allele frequency information to improve its power, thus the performance of the full VAAST 2.0 algorithm is only marginally better than the CASM method alone in this case. Nevertheless, by a small margin, VAAST 2.0 is still the most accurate classifier. At FPR = 0.05, the accuracy of VAAST 2.0 is 4% higher than MutationTaster, the next best classifier.

The variant prioritization accuracies of VAAST 1.0 and 2.0 on the HGMD/1KGP dataset (Fig. 1A and Table1) are very similar. This is because, on this dataset, both algorithms derive most of their power from variant MAF information in a control population. However, in cases where such information is unavailable (e.g., all variants are equally rare), the accuracy of VAAST 1.0 drops, while VAAST 2.0 still accurately predicts the severity of variants using the CASM method. This is illustrated by the *BRCA* variants benchmark dataset in Figure 1B and Table1.

### Benchmark Analyses on Multigenic Common Diseases

Next we compared the power of six aggregative variant association tests using three different published sequence-based disease-gene datasets. The three datasets used are *NOD2*, implicated in Crohn disease Lesage et al., 2002; *LPL*, implicated in hypertriglyceridemia Johansen et al., 2010; and *CHEK2*, a gene involved in breast cancer Le Calvez-Kelm et al., 2011. In the *NOD2* dataset, both rare and common variants are present, while only rare variants (MAF < 0.05) are present in the *LPL* and *CHEK2* dataset. In each study, either genotype or allele frequency data has been reported for diseased and control individuals. In the latter case, genotypes for case and control genomes were simulated, assuming no linkage disequilibrium between variants. Summary statistics for each of the three datasets are presented in Table2. We calculated power using a bootstrap approach. Specifically, we sampled cases and controls with replacement, evaluating the proportion of the resampled datasets that achieved statistical significance Yandell et al., 2011. We used a genome-wide significance level of 2.4 × 10^−6^ for *NOD2* and *LPL*. For *CHEK2*, we set the significance level to 0.0005 for *CHEK2* in concordance with the original study Le Calvez-Kelm et al., 2011.

**Table 2:** Characteristics of the *NOD2*, *LPL*, and *CHEK2* datasets

	Average number of variants per case genome	Number of variants with odds ratio >1	Number of unique multisite genotypes	PAR^a^
*NOD2*	1.19	27	566	44.7%
*LPL*	0.10	10	14	8.4%
*CHEK2*	0.05	22	30	3.81%

a The population attributable risk (PAR) is calculated as the sum of PAR values of all susceptibility variants.

**Figure 2:**
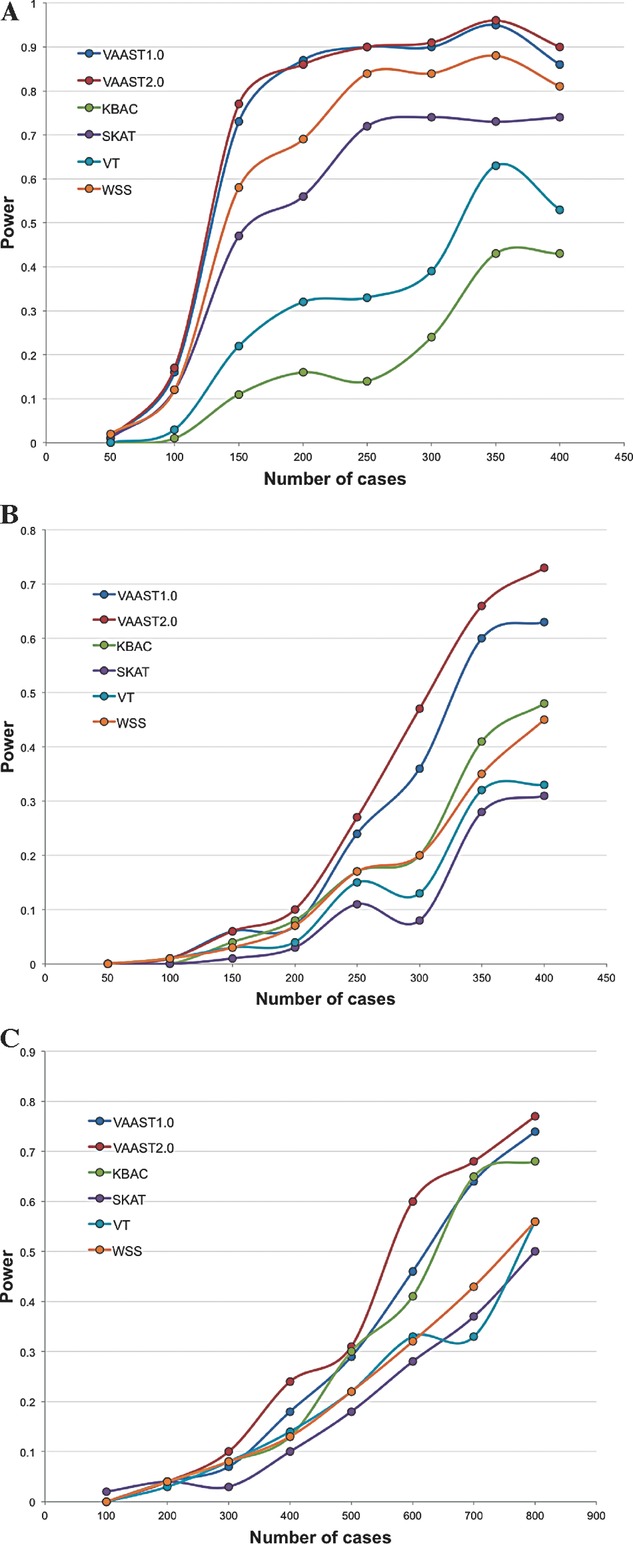
Power comparisons over three published common disease datasets. (A) *NOD2*, (B) *LPL*, (C) *CHEK2*. The *x*-axis shows the number of case genomes and the *y*-axis shows the statistical power. The power is calculated based on 100 bootstraps.

In all three datasets VAAST 2.0 is consistently the most powerful association test (Fig. 2). For *LPL*, for example, at a sample size of 400, VAAST 2.0 has 10% more power than VAAST 1.0 (second) and 25% more power than KBAC (third); For *CHEK2*, VAAST 2.0 has 3% more power than VAAST 1.0 at its maximal sample size and 9% more than KBAC (third); for *NOD2*, the power of VAAST 2.0 is 4% better than VAAST 1.0 and 9% better than WSS (third). Each of the other algorithms seems to have a niche. KBAC, for example, performs well on the two datasets (*LPL*, *CHEK2*) where only rare variants contribute to the disease, but its performance drops significantly where both common and rare causal variants are present (e.g., *NOD2*). WSS, on the other hand, performs well under both scenarios, and outperforms KBAC, SKAT, and VT when common variants are observed (e.g., the *NOD2* data).

We also benchmarked VAAST 2.0 on the Dallas Heart Study dataset Romeo et al., 2009, in which rare variants in *ANGPTL4* gene were found to be associated with low triglyceride levels within 3,551 sequenced individuals. For this study, we tested for different distributions of rare variants in *ANGPTL4* gene between the highest quartile and lowest quartile of triglyceride levels in the 3,551 individuals. Ethnicity and gender status are matched, in accordance with the original study Romeo et al., 2009. For this benchmark experiment, we did not use a bootstrap approach, because the original study did not report the ethnicities and gender information for each individual and as a result we cannot re-create a balanced experimental sampling design using bootstraps. The uncorrected significance values for each test are reported in Table3. All the tests, obtained a *P* < 0.05. Consistent with our other benchmarks, VAAST 1.0 and VAAST 2.0 obtained the lowest *P*-value.

**Table 3:** Significance of associations (showing *P*-values) between low triglyceride levels and rare variants in the *ANGPTL4* gene

VAAST1.0	VAAST2.0	KBAC	SKAT	VT^a^	WSS
0.000371	0.000508	0.00402	0.00677	0.00452	0.00402

a VT is run with PolyPhen-2 scores.

### Benchmark Analyses on Simulated Datasets

Simulated datasets provide an opportunity to investigate the performance of different approaches on datasets presenting specific challenges; for example, under various PARs or under different degrees of allelic heterogeneity, and in a controlled fashion. For these reasons, we used a previously published simulation framework Madsen and Browning, 2009 to compare the power of six aggregative variant association tests (see Supporting Information for details).

**Figure 3:**
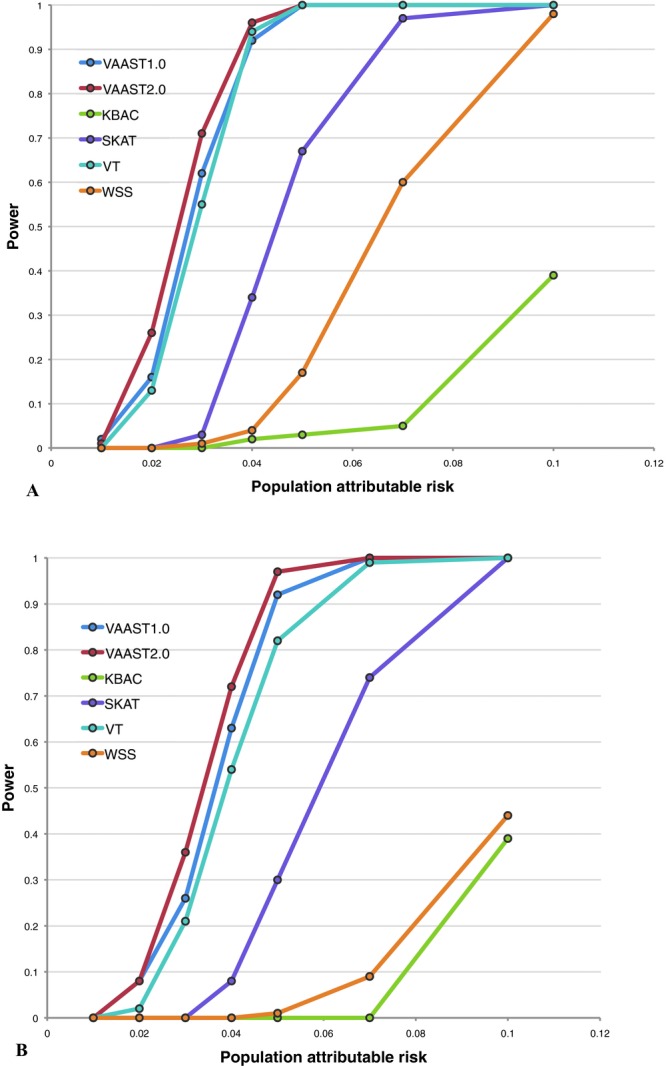
Impact of PAR. Shown is the power of six association tests under different total population attributable risk (PAR) levels. *x*-axis shows the total PAR values from all contributing variants; *y*-axis shows the statistical power based on 100 bootstraps. (A) Dominant model, (B) recessive model. The number of cases and control are set at 1,000, with the number of disease-causal alleles and noncausal alleles both fixed at 50.

**Figure 4:**
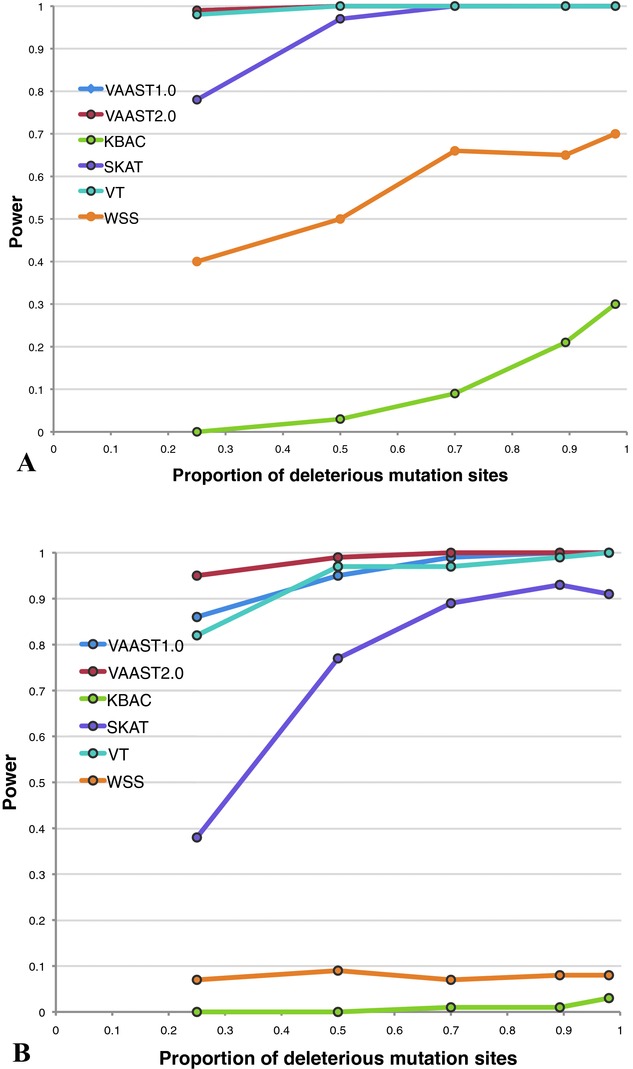
Impact of different proportions of deleterious mutation sites contributing to the disease risk. *x*-axis is the proportion of deleterious mutation sites among all simulated sites; *y*-axis statistical power. (A) Dominant model; (B) recessive model. Total PAR is fixed at 10%; the numbers of case/controls are set at 500; the number of casual variants is 50 with varying number of noncasual variants.

We first benchmarked the power of these tests under different aggregated PAR Madsen and Browning, 2009 values, which reflects the aggregated disease risk of all simulated mutations. These results are shown in Figure 3. Under a dominant model, VAAST 2.0 rapidly achieves 80% power with PARs less than 0.04, and achieves a power of 100% when PAR = 0.05. The power of VAAST 2.0 is followed by VAAST 1.0 and VT, both of which exhibit 10–15% lower power than VAAST 2.0 before reaching 80% power. In contrast, SKAT reached 80% power around PAR = 0.06 and WSS after PAR = 0.07. This trend is also seen in the recessive inherence scenario at various PARs (Fig. 3B). Note that in this experiment we assumed an equal number of causal and noncausal mutation sites, but we also explored other proportions (Fig. 4**)**.

Both VAAST 2.0 and WSS can use user-specified inheritance models (e.g., dominant or recessive) to boost power. However, for the analyses presented in Figure 3, we did not invoke these options, as (1) the other tests have no such functionalities and (2) the mode of inheritance model is not always known. In the published WSS manuscript Madsen and Browning, 2009 where genetic model information is used, WSS achieves 80% power at PAR = 0.05 under the recessive model; in contrast, even without genetic model information VAAST 2.0 has a power of 97% at PAR = 0.05.

**Figure 5:**
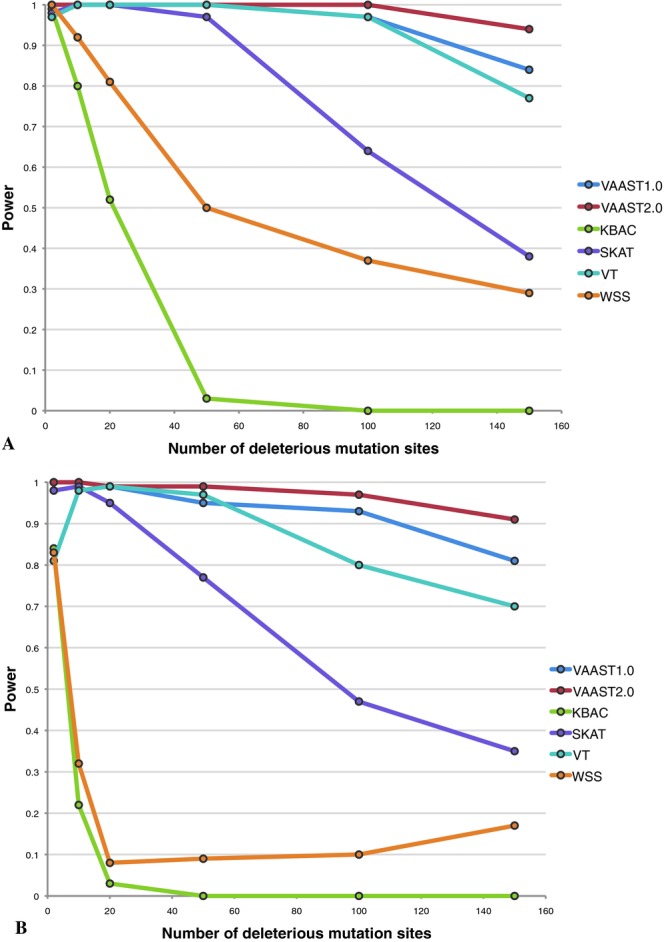
Impact of differing numbers of deleterious mutation sites. *x*-axis is the number of deleterious mutation sites (ND); *y*-axis shows the statistical power based on 100 bootstraps. (A) Dominant model, (B) recessive model. The number of cases and control are set at 500, and the total PAR value is set at 10%.

Next we explored the effect of increasing the number of disease-causal variants (ND) while holding PAR constant in order to model the impact of allelic heterogeneity on the performance of the different algorithms. These results are shown in Figure 5. The actual number of observed causal variant sites per individual is reported in supplementary Table S1. As can be seen, as ND increases, each variant’s risk contribution decreases, along with power. For example, under both dominant and recessive inheritance models, when the number of deleterious variants is 150, each individual variant will only have a PAR of 0.07%. Under this model, both VAAST 1.0 and VAAST 2.0 have greater than 80% power. VT with PolyPhen2 scores seems robust to increasing ND values until ND is greater than 100. For SKAT, the power dropped below 80% between ND of 50 and 100 under dominant model and around 50 under recessive model. KBAC and WSS are less robust to increasing ND than the other methods. We summarize the number of cases/controls required for each algorithm to achieve 80% power in Table4 for ND = 5 and ND = 50.

**Table 4:** Numbers cases and controls required for 80% power in simulations^a^

	Dominant		Recessive	
	ND = 5	ND = 50	ND = 5	ND = 50
VAAST1.0	150	300	300	500
VAAST2.0	150	300	300	400
KBAC	300	>1,000	800	>1,000
SKAT	200	400	300	600
VT	200	300	400	500
WSS	300	700	800	>1,000

a Total PAR is set at 10%.

WSS generally performed quite well, and in many cases outperformed KBAC. We note that the opposite behavior is reported in Liu and Leal 2010. We believe differences in allelic heterogeneity are responsible for this discrepancy. Because KBAC calculates the sample risk for each multisite genotype, in cases where many different casual alleles or common casual alleles are present, the number of multisite genotypes grows rapidly, with a concomitant loss in power. This behavior can be seen quite clearly in Figure 5. Consistent with this hypothesis, KBAC performs well on the *CHEK2* and *LPL* datasets, but does much worse on the *NOD2* data, likely because *NOD2* contains the highest number of multisite genotypes (Table2). We tested this hypothesis by comparing the power of WSS and KBAC under different numbers of deleterious alleles (supplementary Fig. S2, Table S2). When ND = 2 and there are less than 10 multisite genotypes, KBAC has 3–5% more power than WSS before it reaches 80% power. However, as the number of multisite genotypes increases with ND, KBAC gradually looses power, and when there are more than 40 multisite genotypes, the power of KBAC is severely compromised. This result is consistent with its performance on the *LPL*, *NOD2*, and *CHEK2* datasets, suggesting that KBAC is probably best suited for analyses of datasets where the number of distinct multisite genotypes is not large, as demonstrated in Figures 2 and 5.

### Benchmark Analyses on Rare Mendelian Diseases

VAAST was designed to be a general-purpose disease-gene finder capable of identifying both rare and common alleles responsible for both rare and common diseases Rope et al., 2011; Yandell et al., 2011. Although the majority of aggregative variant association tests have been designed for common genetic diseases, there is no a priori reason that they cannot be applied to rare Mendelian diseases. To this end, we benchmarked the six aggregative variant association tests using the benchmarking pipeline from Yandell et al. 2011. Briefly, this pipeline was employed to randomly select 100 Mendelian disease causal genes from the OMIM database, where each gene has at least six disease-causal variants. For each of these genes, we inserted published, disease-causing variants into from one to three healthy Caucasian genomes sequenced on the Complete Genomics platform Drmanac et al., 2010 in order to simulate diseased individuals. For the dominant scenario, we inserted a different single allele into each case genome; for the recessive scenario, we inserted two different alleles into each case genome.

All protein-coding genes are ranked according to the significance of associations between genotypes and dichotomous disease phenotypes. To our knowledge this is the first time that a benchmark of aggregative variant association tests has been conducted on rare Mendelian diseases.

**Figure 6:**
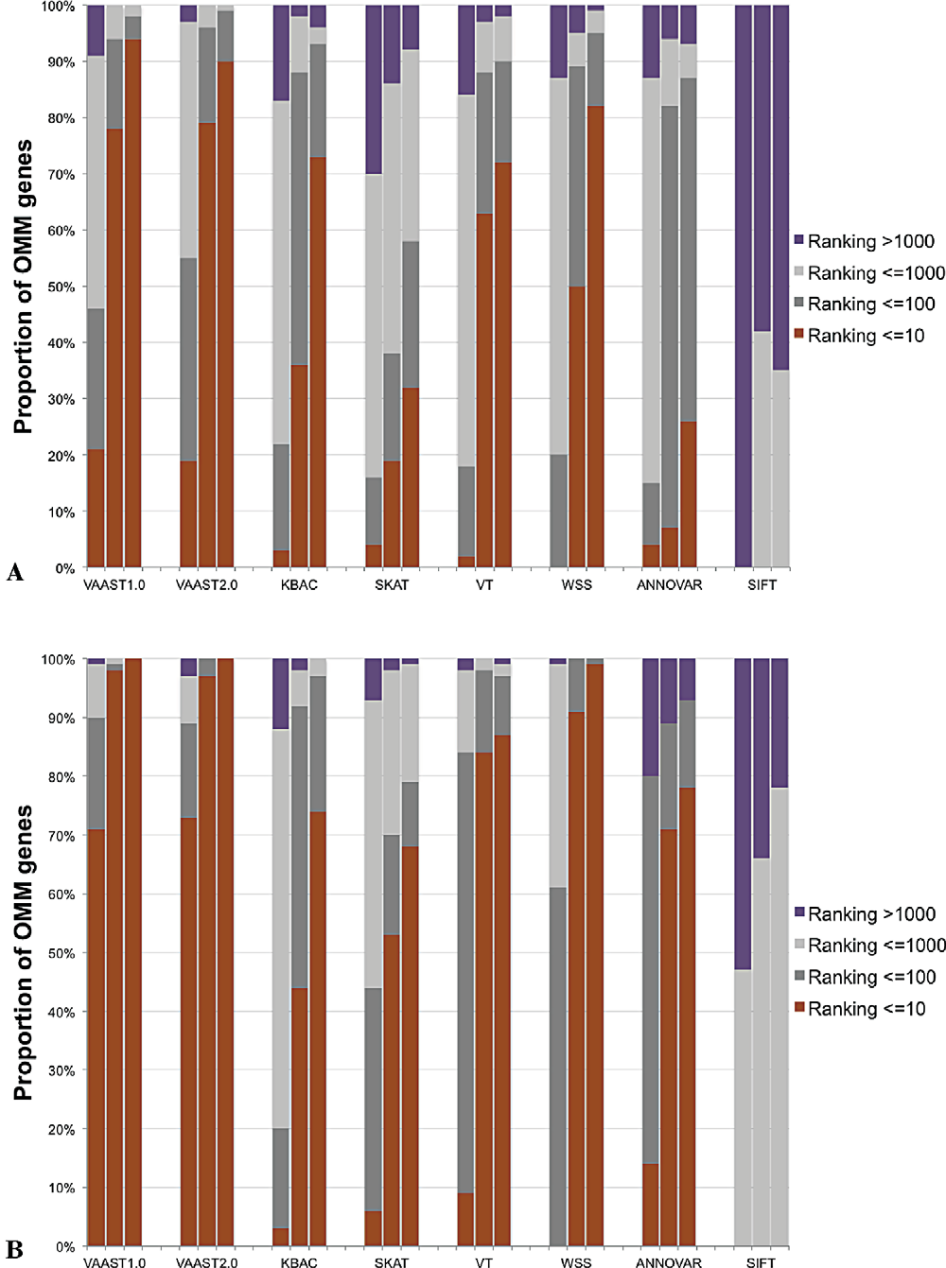
Rankings for 100 different genome-wide searches for known rare disease genes. Panels (A) and (B) shows dominant and recessive models, respectively. The different colors denote the proportion of the 100 OMIM "target" genes falling into four bins based upon genome-wide rank (see insert legend), with orange, denoting the percentage cases for which the disease gene was ranked among the top 10 candidates genome-wide. Dominant and recessive disease scenarios are investigated separately. To model the dominant diseases, one causal variant was inserted into the gene of interest, and in the recessive cases two different alleles are inserted (per case genome). For each algorithm, three columns are shown, corresponding one individual, two individuals, and three individuals as cases.

The results are shown in Figure 6. Figure 6 reports the proportion of the 100 OMIM “target” genes falling into four bins based upon rank; these are bin A: 1–10, bin B: 11–100, bin C: 101–1,000, and bin D: greater than 1,000 among all protein coding genes. Supplementary Figure S3 reports the mean values for these same analyses.

For the dominant disease scenario, with only one case genome (one individual), VAAST 2.0 ranked 19% of disease genes among the top 10 candidates genome-wide and 55% as top 100 candidates. Performance improved dramatically as the number of case genomes increases. With only two case genomes, 79% of disease genes are ranked within top 10, genome-wide; with three case genomes, 90% of disease genes are among top 10. VAAST 2.0’s performance is even better under recessive model. For example, with only one case genome 73% of the disease genes are ranked among top 10, and with two cases, 97% of the disease genes ranked among top 10. We note that in this benchmark analysis, the performance of VAAST 2.0 is very similar to VAAST 1.0 in most cases, suggesting that the CASM approach improves performance primarily on datasets containing common causal variants or complex disease cases.

One of the most intriguing aspects of this analysis is the general finding that most association tests do well on these datasets, despite the fact we included common nonsynonymous SNVs in this analysis, which is expected to violate the underlying hypothesis of many burden tests. For example, using top 10 ranking as an empirical significance level with a sample size of 3, under the dominant model, VAAST 2.0 achieves 90% power, WSS 82%, and KBAC 73% power. Under recessive model, VAAST 2.0, achieves 100% power, WSS 99%, and KBAC 74% power. These analyses thus make it clear that some aggregative variant association tests are excellent rare Mendelian disease-gene finders (e.g., WSS, VT, and KBAC), despite having been developed for common, multigenetic diseases. For purposes of comparison, we also assayed the performance of SIFT and ANNOVAR for rare disease disease-gene identification Ng and Henikoff, 2006; Wang et al., 2010. As would be expected, SIFT does poorly compared to the other tests. ANNOVAR, as a state-of-art approach for the identification of rare Mendelian disease genes, compares favorably to SIFT but in general does not perform well relative to association tests (see supplementary Fig. S3). For example, the power with only one recessive case using VAAST 2.0 and ANNOVAR are 73% and 14%, respectively. This indicates filtering based approaches are suboptimal for the identification of rare Mendelian disorders, when compared to more sophisticated rare-variant association tests. This is true even when the case sample size is as small as one genome.

### Inflation of FPR in Stratified Populations

A potential problem with disease-gene discovery using rare-variant association tests is that if cases and controls are sampled from a stratified population, an inflation of the FPR can result if the proportion of samples from each subpopulation differs between cases and controls Nelson et al., 2012. This situation can occur for a variety of reasons, including biased sampling procedures and differences in disease incidence rates among subpopulations. To explore the behavior of rare-variant association tests when cases and controls are not equally stratified, we simulated case-control data for 1,000 cases and 1,000 controls from the 202 drug target genes presented in Nelson et al. 2012. We evaluated three scenarios: cases and controls from a balanced mixture of two subpopulations (western and northwestern Europeans); modest levels of imbalance between cases and controls (ratio of western to northwestern European genomes of 3:7 in controls and 7:3 in cases), and high levels of imbalance (ratio of 1:9 in controls and 9:1 in cases). As expected, we did not observe an inflation of the FPR in the balanced scenario. Most tests behaved reasonably well when the imbalance between controls and cases was modest, but all tests suffered from substantial FPR inflation when the imbalance was high. SKAT can incorporate demographic information as a covariate matrix, which eliminated the population stratification problem (supplementary Fig. S4). In theory, KBAC can incorporate a covariate matrix as well, although this functionality is not yet available in the KBAC public release. VAAST does not currently provide a solution for this issue. These results indicate that a careful examination of potential population stratification issues is warranted in large studies.

## Discussion

Phylogenetic conservation is a valuable source of information for distinguishing between benign and disease-causing variation. Determining the best way to make use of this information—for purposes of variant prioritization and for association testing—is however, still an open question. Variant prioritization tools, such as SIFT Ng and Henikoff, 2006, use multiple alignments of homologous proteins and judge a human variant damaging if it alters a highly conserved amino acid. PolyPhen-2 goes one step further, making use of protein structural information where available Adzhubei et al., 2010. Because SIFT and PolyPhen-2 rely on conservation information, they can only score variants at conserved positions. VAAST 1.0 implemented a different approach. Rather than evaluating individual columns of multiple alignments in order to judge the impact of a coding variant, VAAST 1.0 used the global, genome-wide frequency of observing a given AAS in any gene, anywhere in the genome. In this regard, the AAS matrix in VAAST 1.0 shared some similarity to Grantham’s matrix Grantham, 1974, which quantifies the property differences between amino acid changes, except that VAAST 1.0 empirically estimated the likelihood of being deleterious or neutral for every AAS using known disease-causing alleles. This means that VAAST could score every coding change, regardless of whether or not a particular gene, or that a particular region of its protein is conserved. Although this approach casts a wider net, VAAST 1.0 was unable to take advantage of position-specific conservation information. Thus, the basic motivation of this work has been to preserve the ability to provide informative scores for every coding variant in the genome while taking advantage of the detailed information provided by multiple sequence alignments wherever they are available. CASM evaluates the global frequency of AAS in the whole proteome, and thus is still capable of making an inference even if there are few or no local alignments. Although the PhastCons scores at these positions are less informative, CASM can still infer severity from type of the amino acid change. As Figure 1 demonstrates, the CASM approach provides an effective solution to this problem, granting VAAST 2.0 a significant advantage in variant prioritization compared to other state-of-the-art tools.

VAAST 2.0, however, is more than a tool for variant prioritization; it is also a tool for genome-wide searches. As such, VAAST is one of several aggregative variant association tests published in the last few years Li and Leal, 2008; Liu and Leal, 2010; Madsen and Browning, 2009; Morgenthaler and Thilly, 2007; Price et al., 2010; Wu et al., 2011. Although several benchmarks have been published Ladouceur et al., 2012; Liu and Leal, 2010; Madsen and Browning, 2009; Price et al., 2010; Wu et al., 2011, ours is the first to systematically compare the performance of these methods across heterogeneous disease datasets—both real and simulated, and for both common and rare diseases. VAAST 2.0 consistently outperforms VAAST 1.0, WSS, VT, KBAC, and SKAT in these analyses, but performance advantages vary across the datasets. Indeed, an important conclusion of our benchmarking analyses is that no single dataset—real or simulated—is sufficient for benchmarking aggregative variant association tests because of the complex behaviors exhibited by these tools. Figure 2 provides an excellent case in point. Collectively, our analyses show how three basic characteristics of case-control datasets impact the performance of the different tools. These are (1) the number of disease-casual alleles; (2) their allele frequencies; and (3) their collective PAR.

The performance curves for KBAC and SKAT serve to highlight the general sensitivity of all the association tests to these three factors. KBAC, for instance, is clearly very sensitive to numbers of deleterious alleles at a given PAR (Fig. 5). Because KBAC estimates the sample risk for each unique multisite genotype, when the number of multisite genotypes is large and each genotype has a relatively low disease risk, the power of KBAC is compromised. KBAC’s poor performance on the *NOD2* dataset, compared to its much better power on the *CHEK2* and *LPL* datasets, further illustrates this behavior. The *NOD2* dataset contains 566 unique multisite genotypes, including a single common variant (MAF 27.7%) that explains 47% of the total PAR of this dataset. In contrast, the *LPL* and *CHEK2* case datasets contain only 14 and 30 distinct genotypes, respectively (Table2) and all of their deleterious variants are rare.

Although SKAT performed well in our simulation studies, it did less well on the three real datasets. Its performance on the *LPL* and *CHEK2* datasets, for example, suggest that SKAT is not well suited for analyses of datasets having modest numbers of casual variants that contribute to a relatively small total PAR (8.4% for *LPL* and 3.81% for *CHEK2*). To test whether SKATs poor performance on these datasets might be due to the fact that it does not group low-risk rare variants, we used VAAST to group variants in *LPL* and passed this information to SKAT at run-time. This approach improved SKAT’s statistical power, from 31% to 45% at maximal sample size (see supplementary Table S3). The fact that SKAT is a supervised method, requiring users to choose kernels and weights, also presents challenges, as the default parameters can be suboptimal for certain cases. This additional complication is demonstrated, for example, on the *NOD2* dataset. SKAT’s default weight resulted in low power (<40% at sample size of 450) because it severely down-weighted common variants, which contribute to a large proportion of disease risk in this dataset. For this reason we used a beta weight value of (1,1) for SKAT for the *NOD2* data, which greatly improved its performance.

In contrast to the other tools, VT and VAAST, when run on simulated data, exhibited very robust and similar performance across a wide range of PARs and allelic heterogeneities, at varying ratios of disease-causing and neutral alleles, and under both dominant and recessive modes of inheritance (Figs. 5). These strengths likely result from two features both tools share in common. First, they directly compare the variant MAF between cases and controls at each site to weight variants. Second, they make use of external predictors of variant function to improve power Price et al., 2010.

Despite their similar performance characteristics on simulated data, VAAST outperformed VT on the real datasets. One possible explanation for this fact is that VAAST 2.0 employs a more flexible variant-weighting method, one that does not rely on a priori assumptions about variant severity and MAF. VT, in contrast, assumes that less frequent variants are more likely to be deleterious, and that a single optimal MAF threshold exists. It thus explores all possible thresholds to find the MAF that maximizes the contrast between cases and controls Price et al., 2010. This assumption is valid for our simulated datasets, but not necessarily true for the real disease dataset. Consistent with these observations, VAAST is the best overall performing tool on every dataset—simulated and real—demonstrating that VAAST 2.0 can cope effectively with the diverse parameter spaces that characterize real case-control datasets.

One limitation of VAAST is that it is currently only supports the analysis of dichotomous traits in a case-control study design, while WSS, VT, KBAC, and SKAT support quantitative traits. For continuous phenotypes, it is possible to categorize samples into two groups (perhaps using only phenotypic extremes), although doing so will often compromise statistical power.

With the exception of VAAST, which has been shown effective for rare-disease gene searches as well Rope et al., 2011, the aggregative variant association tests benchmarked here were developed to identify genes involved in common disease. Our analyses demonstrate that these tests are also applicable to the identification of rare Mendelian disease genes. For example, WSS, VAAST 2.0, and KBAC ranked the disease gene in the top 10 genes genome-wide 99%, 100%, and 74% of the time, respectively, using only three case genomes under a recessive model. The performance compares very favorably to traditional filtering-based approaches, suggesting the necessity of employing association tests in these scenarios.

## Conclusions

Collectively, our analyses illustrate the unexpectedly complex performance characteristics of aggregative variant association tests. They also demonstrate that VAAST 2.0 is a powerful disease-gene finder that performs robustly across a wide variety of simulated and real-world case-control datasets.

## Software and Data Access

VAAST 2.0 is available for download at http://www.yandell-lab.org/software/vaast.html with an academic user license.

## Notes

Disclaimer: Supplementary materials have been peer-reviewed but not copyedited.

**Figure S1** Sample *P*-value versus quantiles of the uniform distribution when the null hypothesis is true (the genotype is not associated with the disease phenotype). **Figure S2** Power comparison between KBAC and WSS, with different numbers of causal variant sites (ND). **Figure S3** Means of the genome-wide rankings of known disease genes in 100 searches. **Figure S4** Distribution of *P*-values when cases and controls are not equally sampled from subpopulations. **Table S1** The average number of observed disease-causal mutations per individual in simulations. **Table S2** The average number of multisite genotypes in the KBAC-WSS comparison. **Table S3** The improvement of the power of SKAT on *LPL* dataset after applying variant grouping.
