# Supplementary material for: VAAST 2.0: Improved Variant Classification and Disease-Gene Identification Using a Conservation-Controlled Amino Acid Substitution Matrix
Source: Genet Epidemiol. 2013 Jul 8;37(6):622–34. doi: 10.1002/gepi.21743 (PMC3791556; doi:10.1002/gepi.21743)
Supplement: Supplementary file 1 — Figure S1 Sample P-value versus quantiles of the uniform distribution when the null hypothesis is true (the genotype is not associated with the disease phenotype). Figure S2 Power comparison between KBAC and WSS, with different numbers of causal variant sites (ND). Figure S3 Means of the genome-wide rankings of known disease genes in 100 searches. Figure S4 Distribution of P-values when cases and controls are not equally sampled from subpopulations. Table S1 The average number of observed disease-causal mutations per individual in simulations. Table S2 The average number of multisite genotypes in the KBAC-WSS comparison. Table S3 The improvement of the power of SKAT on LPL dataset after applying variant grouping. [file gepi0037-0622-sd1.doc]

**Supporting Methods**

**Benchmarking VAAST 2.0 to other variant prioritization tools**

In order to benchmark VAAST 2.0 as a variant prioritization tool, we used disease variants from the HGMD database and variants from 800 genome variant files from 1000 Genome Project . Note that in both sets we selected only variants causing amino acid substitutions or stop codon gain/loss. We first randomly selected the 400 of these genome variant files for training (training-set 1). Because VAAST 2.0 uses a control genome set (which we refer to as the background) as an input to improve its accuracy, the remaining 400 genome variant files not included in training-set 1 were split into two sets comprised of 350 genome variant files (testing-set 1) and 50 genome variant files (testing-set 2). We randomly selected 10,000 common SNVs (MAF >=0.05) from training-set 1 as the non-damaging variant training set (described in the section above), and another 2,000 randomly selected SNVs (common and rare) from testing-set 2 for testing. We chose only common SNVs for training, because MAF is generally negatively correlated with the strength of purifying selection; thus common SNVs are more likely to be neutral. However, because VAAST 2.0 uses allele frequency as part of its variant prioritization process, if we include only common variants in testing-set 2, the comparison could be biased toward VAAST 2.0; thus we also include rare variants in testing-set 2. The testing and training sets did not have any variants in common, and we removed any variants present in OMIM or HGMD database from the non-damaging training set to minimize the chance of including deleterious variants. Because this filter is imperfect, there may be deleterious variants in the non-damaging training and testing sets and thus the benchmark results are conservative, which is a common limitation in variant classifier benchmark studies.

Similarly, disease-causal SNVs from the HGMD database were split into two sets with their size ratio being approximately 9:1. The first set (about 44,000 variants) was used for training and the second set (about 5,100 variants) was used for testing. These two sets also do not have any overlap.

We ran VAAST 2.0 over each of the variants in the test set, with the “–g 0” parameter and otherwise default parameters to calculate its score. The “-g 0” parameter disables variant grouping so that the score is an accurate measurement for each variant individually. We benchmarked three others algorithms (SIFT, Polyphen-2 and Mutation-taster), using pre-computed scores downloaded from <http://www.openbioinformatics.org/annovar/annovar_download.html>.

For evaluation of variants in the *BRCA1* and *BRCA2* genes, we used a set of 1,433 genetic variants collected by Easton et al. . Easton et al. calculated odds ratios for breast cancer causality based upon 1) co-occurrence *in trans* with known deleterious mutations; 2) personal/family history of cancer; and 3) co-segregation of disease in pedigrees. In this study, 133 variants were found to have odds of at least 100:1 in favor of neutrality and another set of 43 variants have odds of at least 20:1 in favor of causality (Tables 3 and 4 in ). We used the 143 missense mutations from these two sets for our benchmark analysis.

**Comparing VAAST 2.0 to other aggregative variant association tests**

To benchmark VAAST 2.0, we compared it with four other recently published aggregative variant association test algorithms (WSS, VT, KBAC and SKAT). WSS has been shown to have superior power compared to CMC , and CAST , so we did not include these two tools in our benchmark analyses. We used PolyPhen-2 scores for VT throughout these analyses, since this improved performance . The VAAST 2.0 package provides native support for all of these association tests. Thus VAAST 2.0 users can directly employ WSS, VT, KBAC and SKAT, supplementing them with VAAST 2.0’s many other features to improve performance.

Our benchmark used a previously published simulation framework described by . Briefly, we simulated several scenarios, each controlling for 1) genetic model (dominant or recessive); 2) number of causal variants; 3) number of cases and controls and 4) total population attributable risk (PAR) of the causal variants. All parameters used to generate these datasets are described in . For each scenario, we performed 100 simulations and measured the power of each method according to the proportion of trials reaching a significance level of 0.05/21000=2.4x10^-6^ (assuming approximately 21,000 genes in the human genome).

For our investigations of the impact of PAR on each test’s performance, we assume that each causal variant has the same individual PAR; hence, each deleterious variant’s PAR is the total PAR divided by number of causal variants in the dataset. Importantly, this is not true of real datasets shown in **Figure 2**, and likely is responsible for some of the performance differences between the simulated and these real datasets.

For each casual variant, its PAR value can be converted to an odds ratio (*r*) with the following formula :

where α is PAR for individual variant and *q_U_* is the genotype frequency in the unaffected population. With this equation, rare variants tend to have higher odds ratios than more common variants at the same PAR. As in , we investigated different levels of total PAR and numbers of causal variants.

For each experiment, we also added an equal number of simulated neutral variants to the case datasets, as justified by . The allele frequencies of simulated variants are sampled from the probability density function given by Wright’s formula using parameters for mildly deleterious mutations . In control genomes, the genotypes of simulated variants conform to Hardy-Weinberg Equilibrium. In case genomes, the phenotypes of neutral variants have the same probability density distribution as in the control genomes, but the causal variants occur more frequently, according to their respective genetic model and risk ratio (calculated from corresponding PAR value; see ).

Under the dominant model, both heterozygous and homozygous causal alleles have the same elevated risk level; For recessive cases, we extended the original simulation pipeline in , so that our recessive model comprises both simple recessive cases and recessive set cases, i.e., both homozygous and compound heterozygous genotypes. We thus did not constrain *p_M_* values (the probability that a haplotype contains at least one disease-risk mutation in unaffecteds ). Note that the simulation procedure assumes no linkage disequilibrium for simulated variants ; our benchmarks on real data assess the impact of this factor on performance.

To simulate the PhastCons scores and amino acid changes, which are inputs to VAAST 2.0, we randomly sampled variants from the HGMD database (for causal variants) and from the 1KGP datasets (for neutral variants), and used their PhastCons scores and amino acid changes for our simulated variants. We removed any variants that were included in the training-sets for VAAST 2.0. The Variable Threshold (VT) method can also use external AAS scores (Polyphen-2 scores) to boost its power ; accordingly we also sampled the PolyPhen-2 scores from the HGMD and 1KGP datasets we sampled above and used this information for our benchmarks of the Variable Threshold method.

Benchmark comparisons were preformed using the weighted sum statistics (WSS) and Variable Threshold (VT) methods as implemented in VAAST 2.0 and in accordance with the original publications. The performance of VT within VAAST 2.0 is also compared to the implementation in the plink-seq package and no discrepancies were observed (data not shown). SKAT and KBAC were benchmarked as implemented by the original authors in the R environment (http://code.google.com/p/kbac-statistic-implementation/; http://www.hsph.harvard.edu/research/skat/download/) and run using a wrapper script available in the VAAST 2.0 package. For SKAT, the “linear.weighted” kernel was used because no variant-epistasis effects were simulated.

Although VAAST 2.0 can employ user-specified genetic inheritance models to increase accuracy, most of the other methods have no such functionalities. Thus in this simulation study we did not provide genetic inheritance model information, even though doing so would likely further improve the performance of VAAST 2.0.

To ensure that our simulation pipeline behaves correctly, we checked that the distribution of p-values conformed to a uniform distribution supported on [0,1] when the null hypothesis is true . That is, when there is no association between disease phenotype and genotype. We tested this by setting PAR value to 0 and calculated p-values with VAAST 2.0 in 10,000 simulations, each consisting 1000 cases and 1000 controls and assuming 100 mutation sites exist in the simulated gene. Indeed, the distribution of p-values agrees very well with the theoretical uniform distribution (Figure S1).

**Benchmarking VAAST 2.0 as a rare Mendelian disease gene finder**

To benchmark the ability of VAAST 2.0 to identify rare Mendalian disease, we first randomly selected a known disease gene from OMIM, together with its published disease-causing alleles. We then inserted these alleles at their reported positions into whole genome variant datasets drawn from the Complete Genomics Diversity Panel . The control (background) genomes dataset consisted of a total of 443 genomes, drawn from multiple sources, consisting of (1) low-coverage exome sequencing data from the 1KGP Pilot Phase ; (2) low coverage Danish exome data ; (3) 10 genomes sequenced with various platforms ; and (4) Complete genomics diversity panel genomes . This control dataset thus contains a variety of sequencing platforms and ethnicities, and as such presents a realistic snap shot of publically available genomes. We ran VAAST 2.0 and the other algorithms and recorded the genome-wide rank of the disease gene, repeating the analyses for 100 different known disease genes (described in detail in ). Whenever there is a tie in the p-values of several genes, the lowest ranking is always used for all genes.

Since ANNOVAR does not calculate a p-value for each gene, the rankings are calculated in a different way in ANNOVAR. Briefly, for each genome, ANNOVAR outputs an unranked list of genes that are potentially disease associated. We thus count the number of times ANNOVAR marks a gene as disease associated among all case genomes, and denote this as the ANNOVAR score. For example, if there are 3 case genomes, ANNOVAR needs to run once on each of the genomes. If we observe ANNOVAR marks gene X as potentially disease-associated 2 out of three times, then the ANNOVAR score for gene X is 2. We then rank all genes according to their ANNOVAR scores. If there is a tie in the ANNOVAR scores, then the lowest ranking is always used for all genes, as for the rare-variant association tests.

The command line used for VAAST 1.0 was:

VAAST -k -d 2e6 -o <output ID> -m lrt -iht <dominant/recessive> <feature definition file> <control cdr file> <case cdr file>.

For VAAST2.0, we used the following command line:

VAAST -l <PhastCons score file> -k -d 2e6 -o <output ID> -m lrt -iht <dominant/recessive> <feature definition file> <control cdr file> <case cdr file>.

**Supplemental Figures**


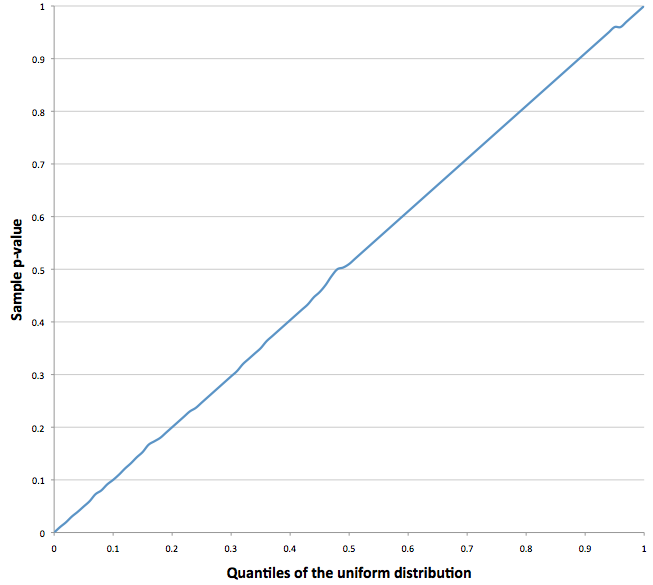


**Figure S1. Sample p-value versus quantiles of the uniform distribution** **when the null hypothesis is true (the genotype is not associated with the disease phenotype).** The PAR is set at 0 and the numbers of cases and controls are both 1000. The distribution of sample p-values is generated from 10,000 simulations.


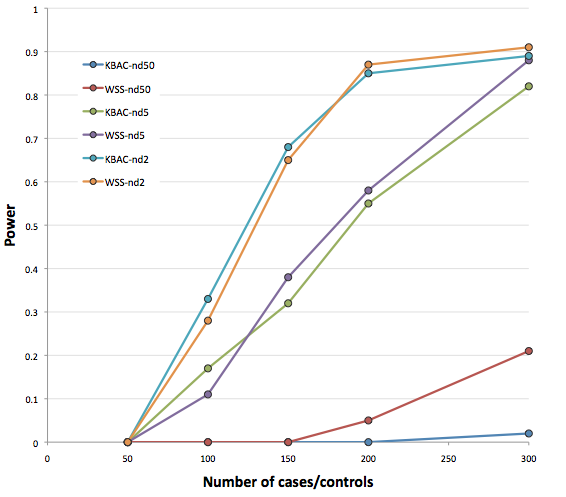


**Figure S2. Power comparison between KBAC and WSS, with different numbers of causal variant sites (ND).** X-axis shows the number of cases and controls and y-axis shows the statistical power. Total PAR is set at 10%. The average numbers of multi-site genotypes for each ND value are reported supplemental table 1.

**
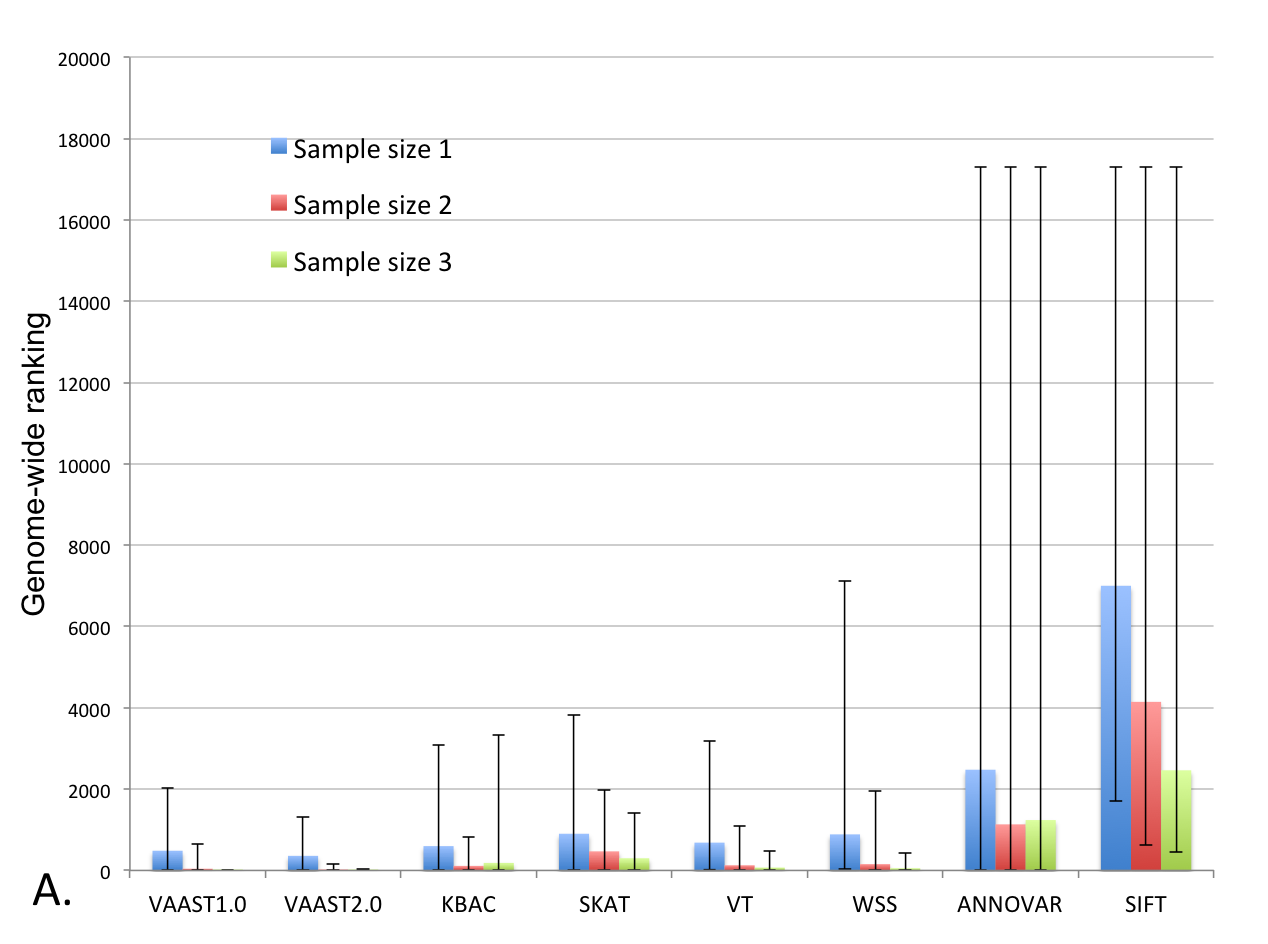
**

**
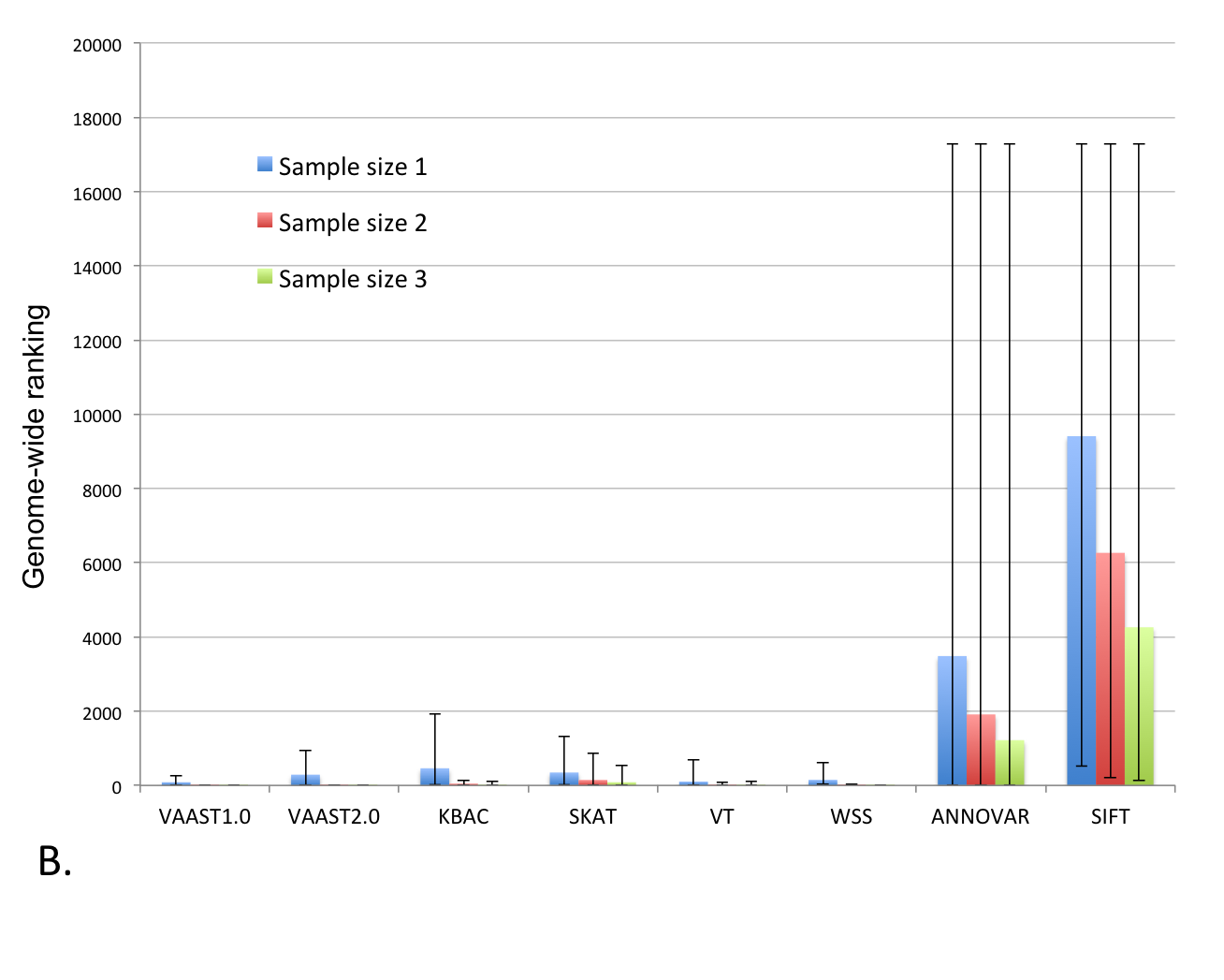
**

**Figure S3. Means of the genome-wide rankings of known disease-genes in 100 searches.** Panel A) and B) shows dominant and recessive models, respectively. Solid bars represent the mean rankings from 100 searches and the error bars represent 95% confidence interval of the rankings. For each algorithm, we show three columns corresponding to sample sizes of 1, 2 and 3.

**
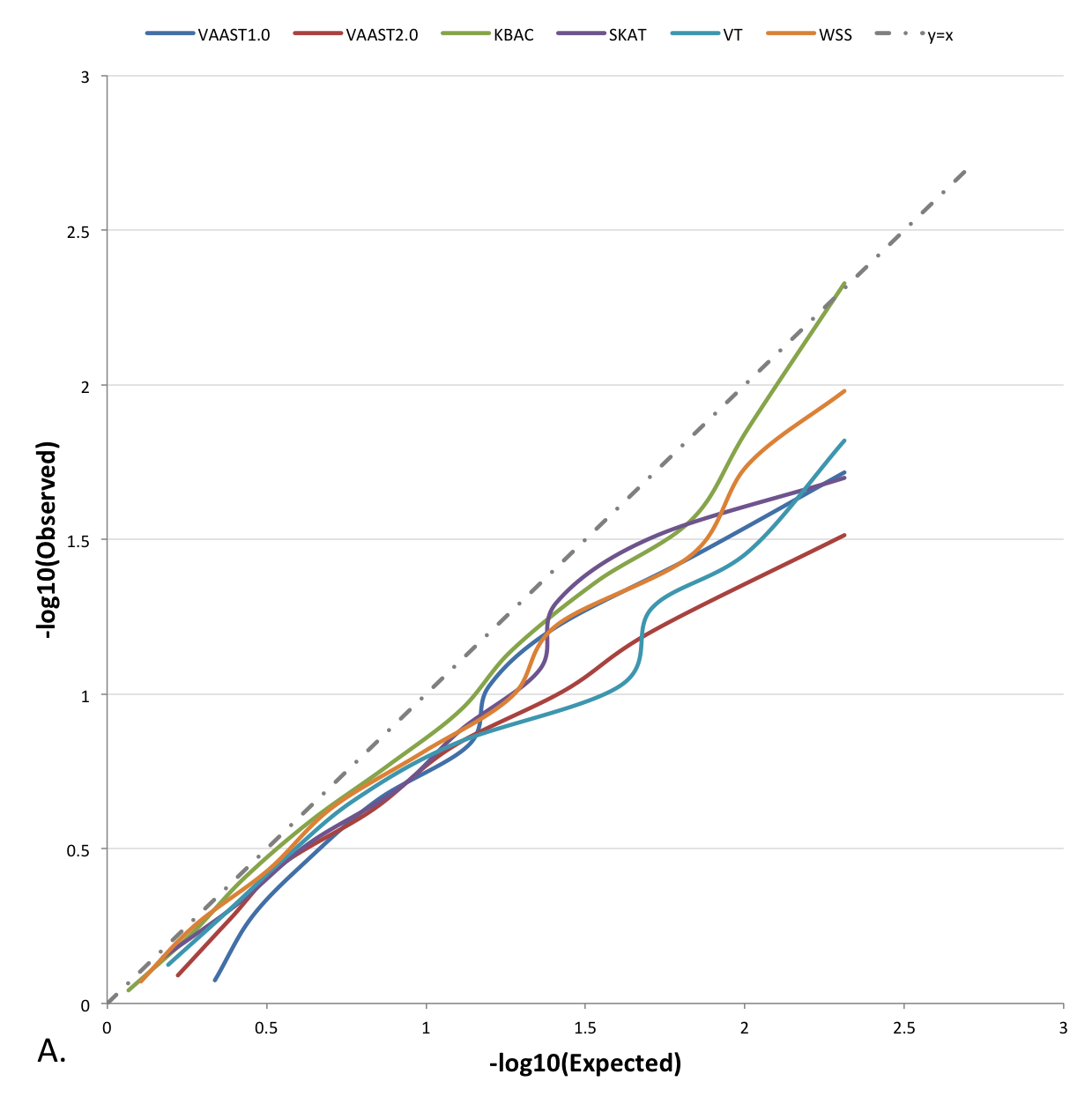
**

**
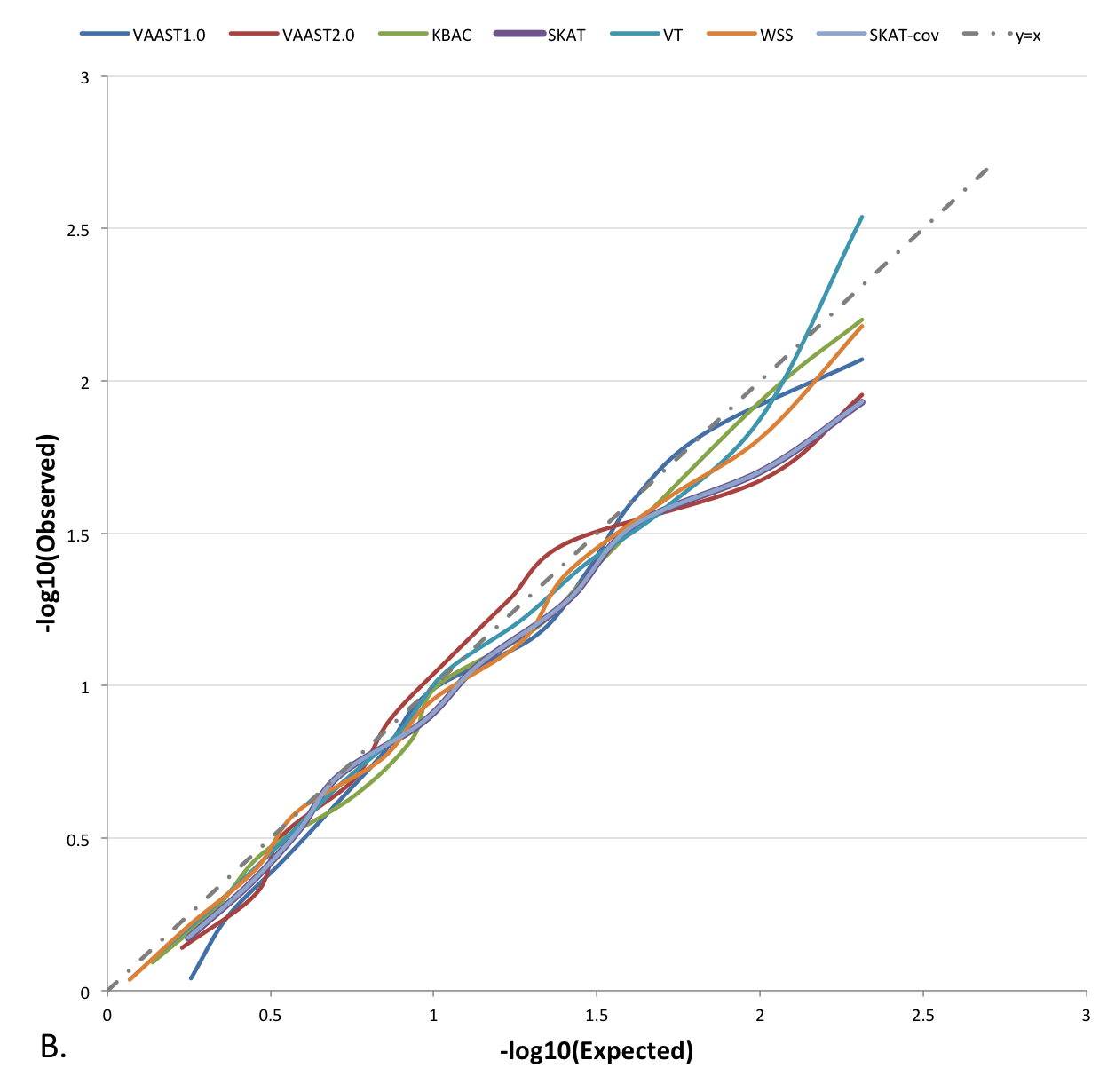
**

**
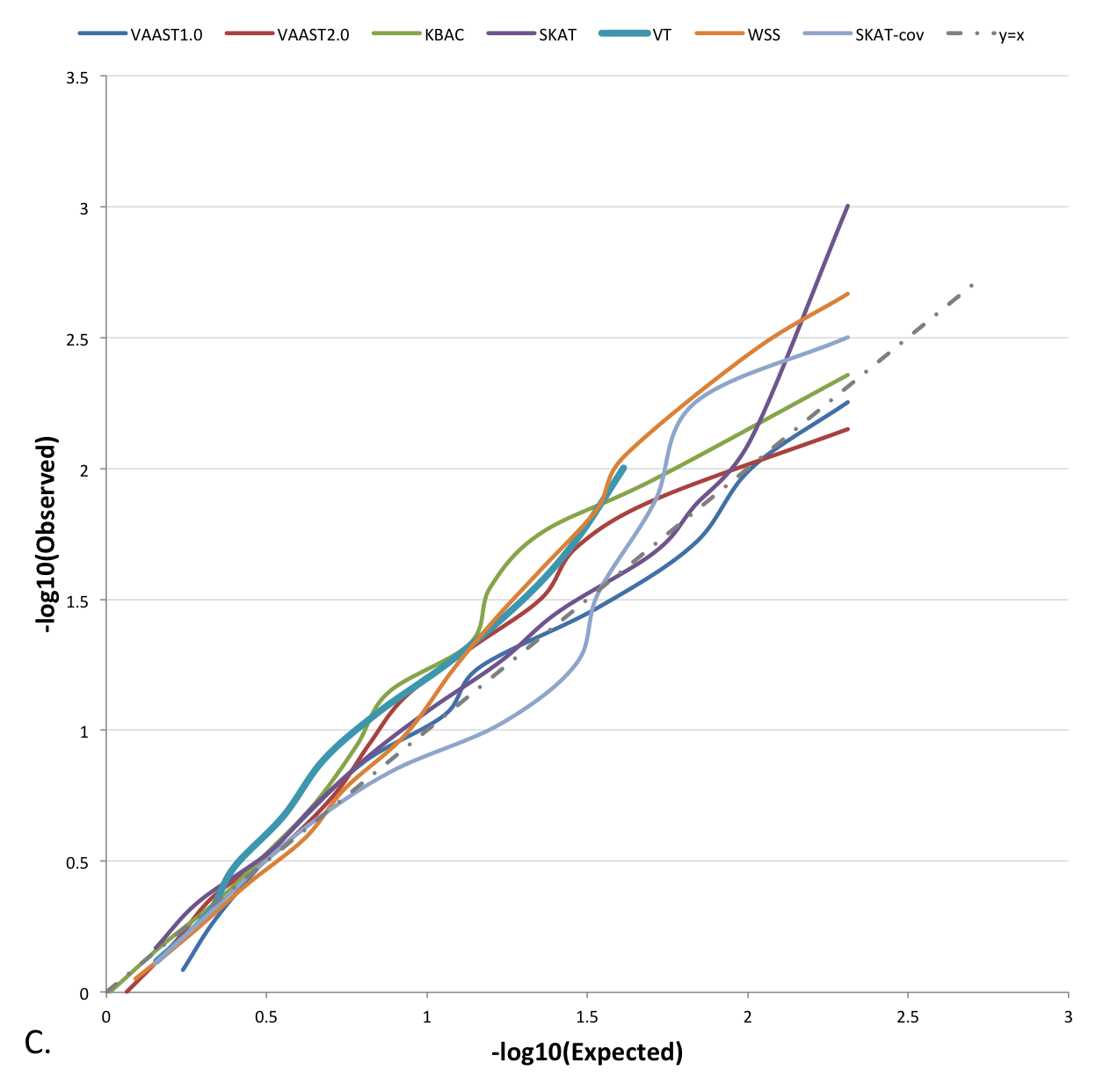
**

**
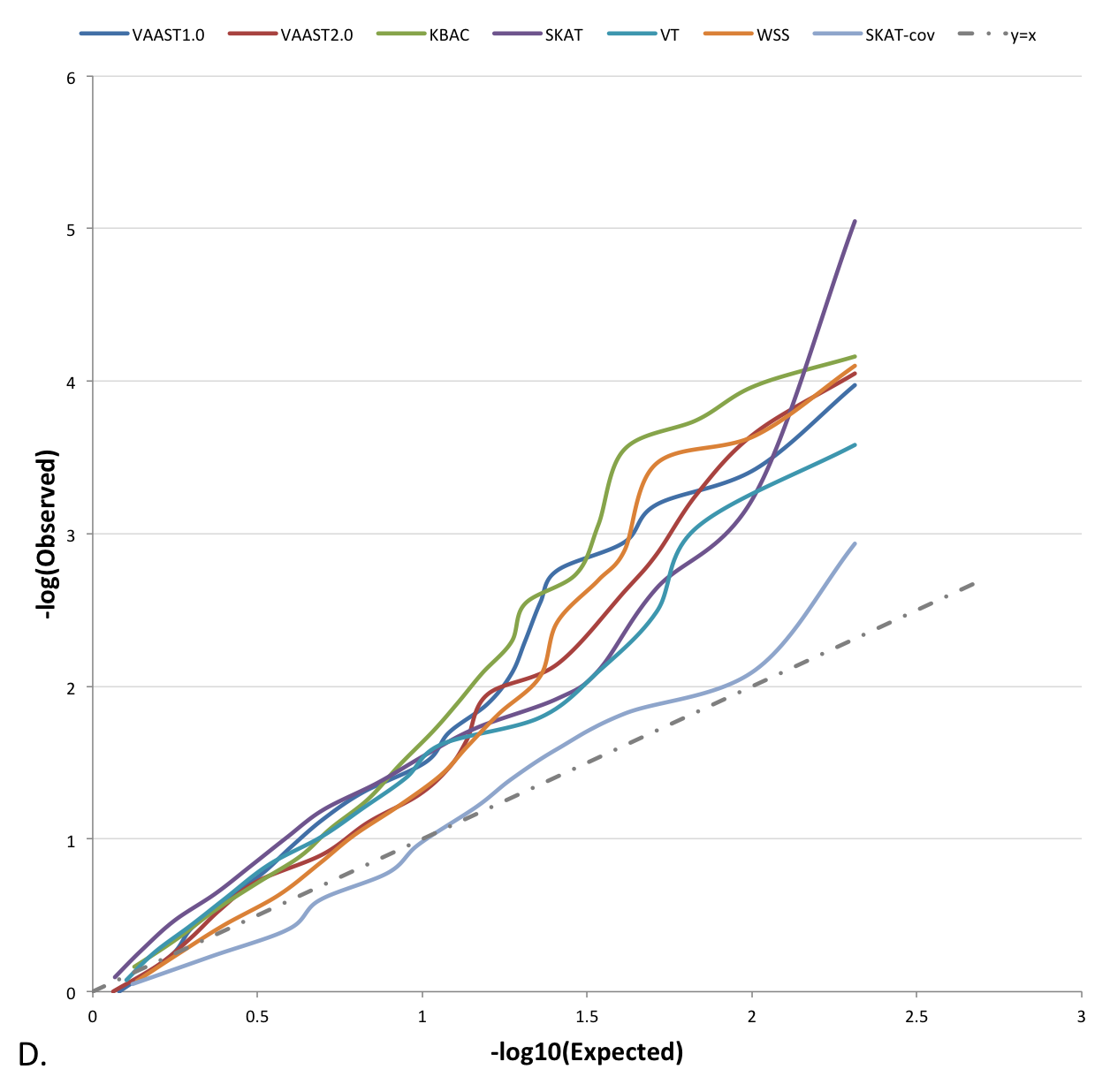
**

**Figure S4. Distribution of p-values when cases and controls are not equally sampled from sub-populations.** A) Both cases and controls are 1000 exomes simulated from northwestern European population; B) both cases and controls are a combination of 500 exomes simulated from northwestern European population and 500 from western European population; C) controls are 300 exomes from western European population and 700 from northwestern European population, and cases are 700 exomes from western European population and 300 from northwestern population; D) controls are 100 exomes from western European population and 900 from northwestern European population, and cases are 900 exomes from western European population and 100 from northwestern population.

**Supplemental Tables**

**Table S1. The average number of observed disease-causal mutations per individual in simulations.** We report the total number of simulated disease-causal mutation sites (ND) in the first row. However, a portion of these variants do not appear in the simulated data because their allele frequency is too low. We thus report the numbers of actually observed causal sites in this table.

| ND | 2 | 10 | 20 | 50 | 100 | 150 |
| --- | --- | --- | --- | --- | --- | --- |
| Dominant | 2 | 10 | 19.96 | 45.08 | 74.6 | 95.26 |
| Recessive | 2 | 9.97 | 19.43 | 41.94 | 70.39 | 93.89 |

**Table S2. The average number of multi-site genotypes in the KBAC-WSS comparison.** Numbers are reported for each number of causal mutation sites being simulated. The sample size is the number of case/control genomes being simulated. See also supplemental figure 2.

| Sample size | 50 | 100 | 150 | 200 | 300 |
| --- | --- | --- | --- | --- | --- |
| nd=2 | 5.93 | 6.81 | 7.69 | 7.91 | 9.15 |
| nd=5 | 9.54 | 13.58 | 16.8 | 18.88 | 18.88 |
| nd=50 | 47.83 | 86.09 | 114.62 | 139.95 | 192.84 |

**Table S3. The improvement of the power of SKAT on *LPL* dataset after applying variant-grouping.** The power of SKAT is reported before and after applying the variant-grouping scheme of VAAST2.0. The significance-level is set at 2.4e-6.

| Sample size | 50 | 100 | 150 | 200 | 250 | 300 | 350 | 400 |
| --- | --- | --- | --- | --- | --- | --- | --- | --- |
| Before variant-grouping | 0 | 0 | 0.01 | 0.03 | 0.11 | 0.08 | 0.28 | 0.31 |
| After variant-grouping | 0 | 0.01 | 0.03 | 0.06 | 0.17 | 0.23 | 0.38 | 0.45 |

**REFERENCES**

1. Cooper DN, Ball EV, Krawczak M (1998) The human gene mutation database. Nucleic Acids Res 26: 285-287.

2. Altshuler D, Durbin RM, Abecasis GR, Bentley DR, Chakravarti A, et al. (2010) A map of human genome variation from population-scale sequencing. Nature 467: 1061-1073.

3. Easton DF, Deffenbaugh AM, Pruss D, Frye C, Wenstrup RJ, et al. (2007) A systematic genetic assessment of 1,433 sequence variants of unknown clinical significance in the BRCA1 and BRCA2 breast cancer-predisposition genes. Am J Hum Genet 81: 873-883.

4. Li B, Leal SM (2008) Methods for detecting associations with rare variants for common diseases: application to analysis of sequence data. Am J Hum Genet 83: 311-321.

5. Morgenthaler S, Thilly WG (2007) A strategy to discover genes that carry multi-allelic or mono-allelic risk for common diseases: a cohort allelic sums test (CAST). Mutat Res 615: 28-56.

6. Price AL, Kryukov GV, de Bakker PI, Purcell SM, Staples J, et al. (2010) Pooled association tests for rare variants in exon-resequencing studies. Am J Hum Genet 86: 832-838.

7. Madsen BE, Browning SR (2009) A groupwise association test for rare mutations using a weighted sum statistic. PLoS Genet 5: e1000384.

8. Wright S (1990) Evolution in Mendelian populations. 1931. Bull Math Biol 52: 241-295; discussion 201-247.

9. Purcell S, Neale B, Todd-Brown K, Thomas L, Ferreira MA, et al. (2007) PLINK: a tool set for whole-genome association and population-based linkage analyses. Am J Hum Genet 81: 559-575.

10. Murdoch DJ, Tsai Y-L, Adcock J (2008) P-values are random variables. The American Statistician 62: 242-245.

11. Drmanac R, Sparks AB, Callow MJ, Halpern AL, Burns NL, et al. (2010) Human genome sequencing using unchained base reads on self-assembling DNA nanoarrays. Science 327: 78-81.

12. Li Y, Vinckenbosch N, Tian G, Huerta-Sanchez E, Jiang T, et al. (2010) Resequencing of 200 human exomes identifies an excess of low-frequency non-synonymous coding variants. Nat Genet 42: 969-972.

13. Moore B, Hu H, Singleton M, De La Vega FM, Reese MG, et al. (2011) Global analysis of disease-related DNA sequence variation in 10 healthy individuals: implications for whole genome-based clinical diagnostics. Genet Med 13: 210-217.

14. Yandell M, Huff C, Hu H, Singleton M, Moore B, et al. (2011) A probabilistic disease-gene finder for personal genomes. Genome Res 21: 1529-1542.
